# Elucidating adolescent aspirational models for the design of public mental health interventions: a mixed-method study in rural Nepal

**DOI:** 10.1186/s13034-017-0198-8

**Published:** 2017-12-21

**Authors:** Sauharda Rai, Safar Bikram Adhikari, Nanda Raj Acharya, Bonnie N. Kaiser, Brandon A. Kohrt

**Affiliations:** 1 Transcultural Psychosocial Organization Nepal (TPO Nepal), Anek Marga, Baluwatar, Kathmandu, Nepal; 2 Duke Global Health Institute, Duke University, Durham, NC USA; 3 Department of Psychiatry and Behavioral Sciences, George Washington University, Washington, DC USA; 4 Department of Psychiatry, Duke University, Durham, NC USA

**Keywords:** Children, Adolescents, Interpersonal violence, Low-income countries, Stigma, Idioms of distress, Cultural models, Substance abuse, Suicide, Nepal

## Abstract

**Background:**

Adolescent aspirational models are sets of preferences for an idealized self. Aspirational models influence behavior and exposure to risk factors that shape adult mental and physical health. Cross-cultural understandings of adolescent aspirational models are crucial for successful global mental health programs. The study objective was elucidating adolescent aspirational models to inform interventions in Nepal.

**Methods:**

Twenty qualitative life trajectory interviews were conducted among adolescents, teachers, and parents. Card sorting (rating and ranking activities) were administered to 72 adolescents aged 15–19 years, stratified by caste/ethnicity: upper caste *Brahman* and *Chhetri*, occupational caste *Dalit*, and ethnic minority *Janajati*.

**Results:**

Themes included qualities of an ideal person; life goals, barriers, and resources; emotions and coping; and causes of interpersonal violence, harmful alcohol use, and suicide. Education was the highest valued attribute of ideal persons. Educational attainment received higher prioritization by marginalized social groups (*Dalit* and *Janajati*). Poverty was the greatest barrier to achieving life goals. The most common distressing emotion was ‘tension’, which girls endorsed more frequently than boys. Sharing emotions and self-consoling were common responses to distress. Tension was the most common reason for alcohol use, especially among girls. Domestic violence, romantic break-ups, and academic pressure were reasons for suicidality.

**Conclusion:**

Inability to achieve aspirational models due to a range of barriers was associated with negative emotions—notably tension—and dysfunctional coping that exacerbates barriers, which ultimately results in the triad of interpersonal violence, substance abuse, and suicidality. Interventions should be framed as reducing the locally salient idiom of distress tension and target this triad of threats. Regarding intervention content, youth-endorsed coping mechanisms should be fortified to counter this distress pathway.

**Electronic supplementary material:**

The online version of this article (10.1186/s13034-017-0198-8) contains supplementary material, which is available to authorized users.

## Background

Adolescent aspirational models influence behavior choices and exposure to risk and protective factors, which ultimately shape adult mental and physical health [1]. Aspirational models are sets of preferences for an idealized self, towards which an adolescent strives, and they are often the reference by which adolescents determine their self-esteem and self-worth [2]. Aspirational models are developed through the interaction of individual experience, local social networks, and exposure to media representations of success [3, 4]. Aspirational models can be applied to recent advances in conceptualizing adolescent interventions in the field of global mental health [5–7].

Effective youth interventions to promote self-esteem and wellbeing are considered best practices in the most recent World Bank guidelines for disease control and prevention (DCP-3). Interventions during adolescence are also associated with life-long positive physical and mental health outcomes [8, 9]. However, there is no one-size-fits-all life course model for youth around the globe, and therefore youth interventions need to be adapted based on local needs, desires, culture, and available resources [8, 10]. There is also a wide variation both between and within countries regarding adolescent mental health, and thus it is necessary to understand local risk and protective factors during adolescence [10, 11].

Research on health and wellbeing of adolescents has increased in recent years. The United Nations Sustainable Development Goals and Global Strategy for women’s, children’s, and adolescent health have pushed this agenda forward [12, 13]. More specifically, there is a need for research on adolescent mental health within low- and middle-income countries (LMICs) [14, 15].

In Nepal, prior studies have explored prevalence rates and risk factors for adolescent mental health problems [16–18]. However, studies have neither addressed how adolescents aspire toward idealized selves nor explored the perceived barriers and resources associated with achieving these goals. We aimed to elucidate adolescent aspirational models in a region of rural Nepal with high rates of adult mental illness [19, 20], with the aim to identify content for mental health interventions.

## Setting

Nepal is ranked among the least developed countries, with a human development index of .54 and per-capita income of 2400 USD in 2014. Per 2011 national census data, children from 0 to 17 years constitute 44.4% of the population of 26.3 million [21]. Political instability, a recent history of violent conflict, structural violence including gender- and caste/ethnic-based discrimination, low quality of infrastructure, limited access to quality education and health services, and lack of employment opportunities are barriers to achieving physical and mental health throughout the country. Although a decade has passed since the People’s War (1996–2006), the country has only recently established a new constitution, which remains highly contentious amid an environment of escalating ethnic disputes, including calls for ethnic federalist redistricting. The fact that almost 1260 people leave the country every day for foreign employment and 24.7% of the gross domestic product is contributed by remittance from these migrants demonstrates the limited in-country resources [22].

The study was set in Jumla, a mountainous district in northwestern Nepal with an area of 2531 km^2^. The district has a population of 108,921, with an average household size of 5.6 [21]. The literacy rate is 55% (male—68% and female—41%), and agriculture is the major occupation. The district is divided into 30 Village Development Committees (VDCs) and has one hospital, the Karnali Academy of Health Sciences Hospital (KAHS), 9 health posts, and 26 sub-health posts. Only 29% of households have access to electricity, and 98% of them use firewood for cooking. Seasonal migration to India is common. Until 2007, Jumla was only connected to the outside world through air travel or three-day walk to the nearest road. Karnali Highway opened in 2007, and though it is functional only during good weather, it has been instrumental in changing the life of people in the district by integrating local, regional, national, and international economies [23].

Jumla’s population is predominantly Hindu (98%). The Hindu caste system, as practiced in Jumla, influences social interactions, life trajectories, and mental health [24]. The caste system in Nepal was formalized by the government through the legal code of 1854, known as the *Muluki* Ain, which divides social groups into high vs. low and pure vs. impure categories. On top of the caste hierarchy are Brahman, the priestly castes, followed by Chhetri/Thakuri castes. Unlike the rest of Nepal, the Chhetri castes in Jumla and surrounding areas are divided into alcohol-drinking *Matwali* and alcohol-abstaining *Tagdari Chhetri* groups. Dalit (previously known as “untouchable”) castes are at the bottom of the Hindu hierarchy [25]. Finally, there are Janajati, ethnic minority groups, the majority of whom are not Hindu [26].

In Jumla, Dalits have been found to have a higher prevalence of depression and anxiety compared to other groups, explained by their low economic status and greater exposure to stressful life events. As in other parts of the world, female gender has been found to be a strong predictor of poor mental health in Jumla [20, 26].

In terms of defining emotions and idioms of distress, some work has already been done in Nepal [27–29]. Definitions are often multifaceted, with common categorizations involving local version of the concepts of heart-mind (Nepali: *man*), brain-mind (*dimaag*), spirit (*saato*), and social status (*ijjat*). Expressions of emotion, especially regarding the brain-mind, are also related to stigma [27]. Alongside these ethnopsychological terms is the use of English terms like “tension” to define emotions [28, 29].

## Methods

The initial phase of the study involved development of interview guides based on previous ethnographic studies, formative interviews with similar populations, and literature reviews of adolescent life choices and burden of mental health problems [30–33]. The first phase of data collection involved a life trajectory interview (LTI) conducted with 20 adolescents, teachers, and parents. This was then followed by a ranking and rating activity conducted with 72 adolescents.

Data collection was completed in collaboration with Transcultural Psychosocial Organization (TPO) Nepal. The first author, a native Nepali with a background in field research and familiarity working in the study site, conducted the initial life trajectory interviews and card sorting activity and trained the other TPO researchers at Jumla. Both other TPO researchers (2nd and 3rd author) had more than 4 years of research experience and training in qualitative and quantitative methods, as well as ethics of research with vulnerable populations. These field researchers were also certified psychosocial counselors and provided first-hand psychosocial counseling to participants whom they screened as having some form of mental health and psychosocial problems. Data collection occurred from September 2014 through May 2015. In this study, adolescents were defined as people from 15 to 19 years of age. The age group was selected because this range captured the cultural notion of adolescent in Nepal [30].

### A. Life trajectory interview (LTI)

The LTI was designed to understand the link between large-scale structural conditions and social processes with individual outcomes. It investigates how life-course models mediate the relationship between adolescent development and later psychiatric conditions [34, 35]. Six themes were included:

1. Understanding the ideal person [*raamro maanche*]
2. Life goals
3. Barriers and resources
4. Emotions and coping
5. Interpersonal conflict
6. Alcohol and suicide.

These six themes were chosen based on prior research in the study site. Because preventing adolescent suicide was a broader aim of our work in Nepal, we prioritized themes related to youth suicide and mental health. Suicide is the single leading cause of mortality among women of reproductive age [36], and in Jumla, the area where this study was conducted, 85% of suicides among women occur before the age of 25 years [37]. Work on suicide and mental health in this region of the country and elsewhere in Nepal has highlighted the importance of alcohol use, interpersonal conflict, thwarted life goals, emotional dysregulation, and lack of coping skills as risk factors [38–40]. The six themes were piloted in four initial interviews conducted jointly by the first and last authors and through ethnographic observation in Jumla.

The *“* ***ideal person*** *”* theme explored the respondent’s understanding of an ideal person. It described the general qualities of an ideal person through an individual, social, and cultural perspective. *“* ***Life purpose and goals*** *”* explored the life purpose of the respondent and the general adolescent population in Jumla. It also explored the similarities and differences in life goals with their parents and ways to balance them. *“* ***Barriers and resources*** *”* looked at the possible internal and external barriers that were likely to occur in their life and the resources to address it. *“* ***Emotions*** *”* looked at the different positive/negative emotions they experience and ways to cope with them. We especially looked at “tension,” which is an English idiom for stress and psychological distress increasingly used in South Asia by both adult and adolescent populations [28, 29]. For “***Coping***,” we wanted to make the distinction between two different themes: sharing feelings (*man ko kura satne*: sharing things in the heart-mind), which is considered a positive behavior by adolescents, and venting/projecting negative emotions onto others (*aru lai rish pokhne*: throwing anger onto someone else) as a dysfunctional way of channeling feelings. “***Interpersonal conflicts***” explored difficult and abusive social relationships. “***Alcohol, substance use, and suicide***” addressed substance use attitudes and behaviors among adolescents in Jumla.

Each interview took 60–90 min, and a debriefing form was written after every interview. Most interviews were digitally recorded with participant’s consent. Four participants did not provide consent for recording, so detailed notes were taken for those interviews. Of the four not consenting for audio recording, three were adolescents who did not feel comfortable being recorded. One teacher did not consent for recording because of fear that the recording could be obtained by persons other than the researchers. Although not explicitly stated, the history of political violence during the Maoist revolution in the area (1996–2006) may have influenced comfort with audio recordings. In particular, Maoists had targeted teachers leading to particular sensitivity of these participants. Interviews were transcribed directly into English. Coding was done using Nvivo Version 10 using thematic analysis [41]. The first author coded all the interviews with a codebook developed jointly by the first, second, and senior author based on close reading of transcripts.

Altogether, 10 themes and 74 sub-themes were identified, which became the basis for the card ranking and rating tasks. The themes were:

1. Qualities of an ideal person (*Raamro maanchhe*)—8 sub-themes
2. Life goals—8 sub-themes
3. Barriers for life goals—7 sub-themes
4. Resources for life goals—4 sub-themes
5. Positive emotions/thoughts—6 sub-themes
6. Negative emotions/thoughts—7 sub-themes
7. Coping mechanisms—9 sub-themes
8. Causes of violence—9 sub-themes
9. Causes of alcoholism—7 sub-themes
10. Causes of suicide—9 sub-themes.

In accordance with recommendations for transparency and availability of qualitative data while protecting anonymity of participants [42], examples of qualitative coding queries are presented in Additional file 1.

### B. Card sorting (ranking and rating task)

Cultural consensus analysis is a set of techniques used to understand how people in a cultural group make sense of information within a domain [43, 44]. Common methods used in cultural consensus analysis include free listing, ranking, and pile sorts. We employed a modified ranking and rating card sort that allowed for a visual display of preferences, timeline, thoughts, and frequency related to the ten themes identified in the life trajectory interviews [45, 46].

**Fig. 1:**
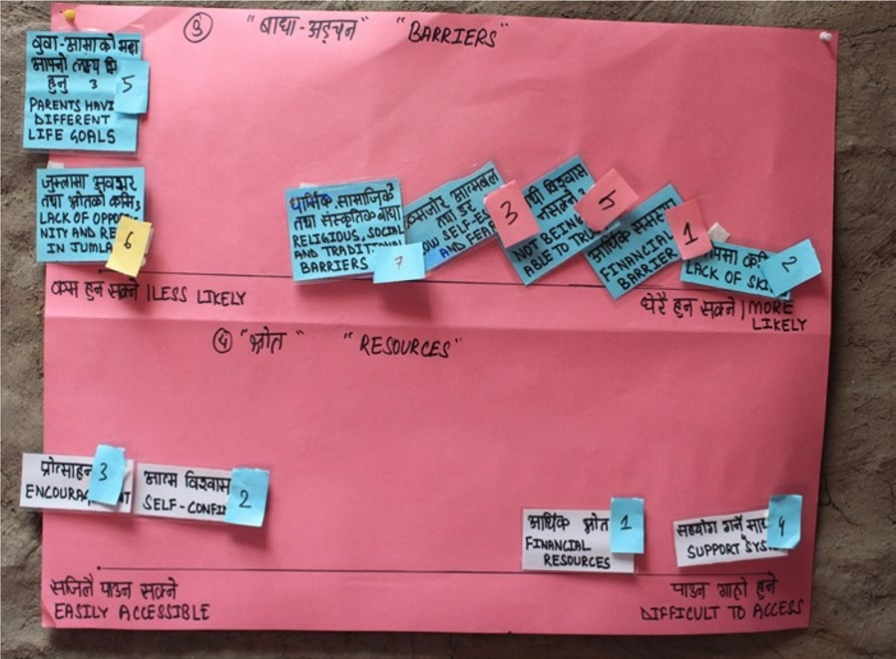
Card sorting example. In the top half of the poster-paper, the respondent places barriers on an axis from less likely to happen (left side of photo) to more likely to happen (right side of photo). The numbers on the items refer to how severe the barrier is. For example, “financial barriers” were ranked #1 (most severe) and very likely to happen (just below “lack of skill”—the most likely barrier). In the bottom half of the poster-paper, resources are sorted from easily accessible (left side) to difficult to access (right side). Numbers on resource items refer to importance. Financial resources were ranked most important and the second most difficult to access

The 10 themes were written on separate sheets of poster paper, and index cards were developed for the 74 sub-themes. For each theme, the participant was given the set of corresponding index cards and was asked to rank the items based on preference, timeline, thoughts and/or frequency. For example, in Fig. 1 the participant was given a set of seven cards, and the respondent first chose the cards that were relevant for their life; this respondent included all cards. Then the respondent ranked the index cards by assigning a number to each card. Finally, the respondent indicated how likely they were to experience those barriers in their life by placing them in the specified area of the chart. Here, keeping the index cards on the left means the items were less likely to happen, and on the right, it meant the items were more likely to happen in their life. They had the choice of discarding cards that were not relevant to them. The charts were then photographed, and scores were entered by overlaying a visual matrix onto the photographs.

Before using this with study participants, the procedure was pilot tested with research staff at TPO Nepal to evaluate its acceptability, feasibility, and comprehensibility.

Ethnicity and gender were the two main demographic factors examined to test associations with ranking and rating data. These two factors were evaluated for associations with the eight themes: quality of an ideal person, life goals, barriers, frequency of emotion, coping mechanisms, causes of violence, alcohol use, and suicide. Demographic factors were tested separately for their significance using one-way ANOVA tests. A statistical significance of *p* < .05 was used. SPSS [Statistical Package for the Social Sciences Version 24 (IBM/SPSS, 2016)] was used for statistical analysis. All quantitative data are available in Additional file 1.

## Results

Twenty respondents participated in the life trajectory interviews (LTIs) (see Table 1). Sixty percent were female. The majority (75%) of respondents were adolescents, and the remaining 15% were teachers and 10% parents. The participants represented the four major castes/ethnicities in Jumla–Brahman (35%), Chhetri (20%), Dalit (30%) and Janajati (15%). Adolescents included both students (n = 10) and youth who had dropped out of school (n = 5).

Seventy-two adolescents participated in the card sorting exercise, with equal representation of boys and girls. As it was necessary for the participants to read the index card and charts, only school-going or literate adolescents were selected. One-third of the ranking sample was high-caste (Brahman or Chhetri), one third was low caste Dalit, and one third was Janajati (Table 1).

**Table 1:** Sample characteristics

	Life trajectory interviews (n = 20)	Card sorting (n = 72)
	n (%)	n (%)
Gender		
Male	8 (40%)	36 (50%)
Female	12 (60%)	36 (50%)
Caste		
Brahman	7 (35%)	13 (18.1%)
Chhetri	4 (20%)	12 (16.7%)
Dalit	6 (30%)	23 (31.9%)
Janajati	3 (15%)	24 (33.3%)
Group		
Adolescent	15 (75%)	72 (100%)
Teacher	3 (15%)	–
Parent	2 (10%)	–

### Qualities of an ideal person

An ideal person (Nepali: *raamro maanche*) was someone whom respondents aspired to be. LTI responses included attributes for thoughts, behaviors, education, and physical features. More than half of respondents reported education to be the most important characteristic of an ideal person (Table 2). Among the four caste groups, Dalit adolescents saw “socially acceptable behavior” as the most important character of an ideal person, and Brahmans saw it as the least important (caste/ethnicity group difference ANOVA, *F* = 4.25, *p* = .008). In contrast, Brahman adolescents endorsed being physically healthy and good looking as the most important characteristic (*F* = 3.99, *p* = .011).

**Table 2:** Card sort results (n = 72)

	Rank^a^	Mean	SD	Gender		Ethnicity	
				F	*p* value	F	*p* value
*Qualities of ideal person*	(1–8)						
Educated	1	6.80	1.80	.61	.43	.64	.59
Socially acceptable behavior	2	5.46	1.50	.50	.48	4.25	.008**
Positive thinking	3	4.65	2.30	1.15	.29	.21	.89
Helping others	4	4.49	2.44	.84	.36	.84	.48
Keeping family happy	5	4.13	2.48	.11	.74	1.36	.26
Self-satisfaction	6	3.92	2.19	.01	1.00	.39	.76
Physically healthy/good looking	7	2.87	2.72	2.34	.13	3.99	.01*
Religious	8	2.73	2.45	.29	.59	.51	.68
*Life goals*	(1–8)						
Better education	1	7.42	1.13	.68	.41	7.49	.001**
Getting a job	2	6.17	1.24	3.10	.08	3.81	.01*
Following your dreams	3	5.65	1.76	2.83	.09	.55	.65
Earning money	4	5.07	1.57	.44	.50	1.30	.28
Business	5	3.77	2.09	.01	.92	1.75	.16
Migration	6	3.48	1.91	.06	.43	.72	.55
Continue family tradition	7	3.48	1.50	4.14	.05*	1.17	.33
Marriage	8	2.73	1.53	.41	.52	1.17	.33
*Barriers*	(1–7)						
Finance	1	5.46	1.88	2.50	.12	1.28	.29
Low self-esteem	2	4.59	1.91	.13	.72	.75	.52
Skills	3	4.57	1.94	.09	.75	1.46	.23
Lack of opportunity	4	4.18	1.48	1.85	.18	.53	.66
Trust	5	3.68	2.04	.31	.58	.81	.49
Different life goals with parents	6	3.51	1.92	.38	.54	.89	.45
Religious/cultural barriers	7	3.18	2.01	1.16	.29	2.40	.07
*Frequency of emotions*	(1–7)						
Tension	1	6.00	2.34	5.27	.03*	1.98	.12
Sadness	2	4.49	2.23	2.01	.10	.33	.80
Embarrassment	3	4.25	2.77	.16	.68	.75	.52
Anger	4	4.01	2.74	.01	.89	1.31	.28
Inferiority	5	3.71	2.31	1.62	.21	1.32	.27
Fear	6	3.70	2.72	.85	.36	.19	.89
Self-guilt	7	3.36	2.72	.02	.87	.19	.90
*Coping mechanism*	(1–9)						
Sharing	1	6.08	2.17	.10	.70	2.84	.04*
Self-consoling	2	6.08	2.01	.21	.06	.42	.74
Thinking continuously	3	4.37	2.38	.03	.60	.55	.65
Staying alone	4	4.31	2.36	1.96	.16	4.73	.005**
Crying	5	3.85	2.49	.003	.96	.91	.44
Venting	6	3.77	1.96	3.99	.05	.82	.49
Acceptance	7	3.65	2.45	4.56	.04*	1.17	.32
Alcohol	8	1.58	2.16	.03	.85	1.43	.26
Suicidal thoughts	9	1.09	1.84	.17	.68	1.93	.15
*Causes of violence*	(1–9)						
Bad habits	1	6.75	2.33	1.25	.27	.12	.94
Alcohol	2	6.26	2.65	1.26	.26	1.35	.26
Financial issues	3	6.10	2.29	.66	.42	1.76	.16
Less coping/tolerance	4	5.52	2.63	.56	.46	3.49	.02*
Inequality	5	4.89	2.22	1.14	.29	.86	.46
Misunderstanding	6	4.86	2.22	.39	.53	.46	.71
Culture/traditional reasons	7	4.73	2.19	.09	.76	.37	.77
Not obeying parents	8	4.47	2.15	1.40	.24	.62	.60
Unhealthy competition	9	3.70	2.64	1.88	.17	.54	.65
*Reasons for alcohol use*	(1–7)						
Tension	1	6.29	2.04	8.07	.006**	.29	.83
Friend circle	2	5.93	2.16	.07	.79	.98	.40
Family environment	3	4.34	2.78	.09	.77	.84	.47
To have fun	4	4.29	2.42	2.30	.13	.96	.42
To relax	5	3.74	2.17	.07	.79	.75	.52
Tradition	6	3.64	2.82	.84	.36	1.11	.35
Cultural traditions	7	3.08	2.52	.31	.58	.99	.40
*Causes of suicide*	(1–9)						
Domestic violence	1	5.65	2.33	.30	.58	.41	.74
Love tragedies (romantic break-up)	2	5.60	2.49	.18	.67	.50	.68
Exam pressure	3	4.94	2.37	2.26	.14	1.22	.31
Alcohol problems	4	4.62	2.62	.16	.69	.57	.64
Financial issues	5	4.57	2.61	.16	.69	1.42	.25
Relationship issues (other than romantic relationships)	6	4.47	2.07	.20	.65	.69	.56
Social status	7	3.18	2.33	.01	.96	.17	.92
Interpersonal conflict	8	2.71	2.13	.29	.59	.77	.51
Lack of social support	9	2.65	2.48	.98	.33	1.24	.30

^a^Lower numbers refer to higher ranking for importance or frequency (e.g., a ‘1’ for qualities of ideal person refers to the highest ranked quality; a ‘1’ for frequency of emotion refers to the most commonly experienced distressing emotion)

* p value: < *.05*, ** p < .001

### Life goals

Results from card sorting and LTIs revealed that the greatest importance was placed upon education and obtaining government jobs. All participants in card sorting chose education as one of their life goals, of which 75% selected it as the most important. Importance of education was highest among Dalit respondents and lowest among Brahman respondents (F = 7.49, *p* = .001). Government jobs, locally termed as “*lok Shewa,*” refers to being a bureaucrat or becoming a police officer or army soldier. Chhetri respondents ranked the importance of government jobs higher than Dalit respondents (F = 3.81, *p* = .013). For example:

*“I want to study a lot first. I want to study up to a higher level, go to different places, understand and learn many things and ultimately become a nurse.”*—15-year-old Dalit Female

*“My child’s first priority is to study, become a great person, stand on her own feet and get married only after she achieves this. I will support this.”* 45-year-old Brahman Parent, Female

Migration and marriage were among the least prioritized life goals. Migration was predominantly a goal for those who wanted to travel within the country to obtain higher education. Migration for work was not prioritized. LTIs revealed that marriage was seen as a goal only after education was completed. Among the five adolescents who had dropped out of school, two of them (1 male and 1 female) were married and had dropped out of school after marriage. Continuing traditional family occupations (e.g., farmer, Hindu Brahman priest, Dalit blacksmith, Dalit cobbler) was the second lowest ranked life goal but was found to be statistically significant, with more girls wanting to continue their family tradition than boys (F = 4.14; p = .047). For example:

*“I have given up trying to convince my parents* [to change their traditional beliefs]*. But when I am menstruating, I do not have to sleep in the cowshed. I can sleep at home but cannot go downstairs, and my parents take me to hospital if I have lot of pain. It is slowly changing.”*—17-year-old Janajati Female

### Barriers to life fulfillment

The greatest barrier in fulfilling life goals came in the form of poor finances and low self-esteem. Financial resources were required for continuing education, learning new skills, and added labor. The participants also noted lack of skills, opportunities, and institutions to continue education as possible barriers:

*“I have written songs and want to record an album, but there is no such opportunity and resources here in Jumla. There is no place to even getting trained in singing, and I cannot go to Nepaljung* [nearest city] *to do all these.”*—18-year-old Dalit Male who was an aspiring singer

Religious and cultural barriers scored the lowest in terms of barriers to achieving life goals.

### Emotional distress

The English-language term ‘*tension’* was the most frequently endorsed negative emotion among the adolescents. As discussed in “Methods” section, this English language term is increasingly used in South Asia to denote stress and psychological distress, whereas other emotional terms were in Nepali. Girls rated the frequency of tension higher than boys (F = 5.27, p = .025). Sadness and embarrassment were also noted as other frequent manifestations of emotional distress:

*“I get* ‘tension’ *when I am practicing my run*—*especially while running up and down the hill. I already have a hearing problem, and I get* ‘tension’ *that there will be no one to take care of me and nothing to do with my life if I fall and break my legs and hands.”*—18-year old Brahman Female

### Coping

Sharing with friends/families and self-consoling were the most common forms of coping mechanism in Jumla. There were caste differences regarding how people coped through sharing. Brahman respondents described sharing the most (F = 2.84, p = .04) and staying alone the least (F = 4.73, p = .005), with Chhetri respondents saying the opposite, staying alone the most and sharing the least. Gender differences were found in expressions of emotion. Boys endorsed more acceptance of their emotions than girls did (F = 4.56 p = .036). For example:

*“Whenever I face difficulties and get negative thought, I share it with my sisters. I also do pooja* [prayers] *and share my happiness and difficulties with God.”*—15-year-old Brahman Female

*“When I am worried, I call my brothers immediately because I suppose they will say something to me and ask why I am feeling like that. I will tell them openly because they have been supporting me for very long. Then my brother convinces me and asks me not to think like that. So, I am always searching for my cellphone to call my brother during times like that.”*—17-year-old Janajati Female

Many adolescents utilized self-consoling to cope with their problems and emotions.

“*When I have bad thoughts, I look at my friends and observe what they do to remove those thoughts. By looking at them I know what I should do to remove them and gets convinced that it is not just me but others too who are having those negative thoughts*.”—15-year-old Chhetri Female

### Causes of violence

Bad habits including gambling and domestic feuds topped the list in major causes of violence in Jumla, followed by alcohol use. Caste differences were noted pertaining to thresholds for physical retaliation with violence. Janajati respondents were found to have the lowest threshold to respond with physical violence when engaged in altercations with others (F = 3.49, p = .021).

*“When people drink alcohol, they use bad and foul language. Those people who are not drunk cannot tolerate someone speaking with a foul mouth to them and then the fighting starts. Drunk people start physically assaulting people they are quarreling with.”*—16-year-old Janajati Male

### Reasons for alcohol use

Tension and a coercive peer and family environment were described as the major causes of alcohol use. Females were found to be more prone to drinking than men because of tension (F = 8.07, p = .006). Cultural drinking practices scored lower for harmful alcohol use:

*“These days people from all caste/ethnicity have started to drink. They drink openly or secretly. There is a liquor store opposite to the place where I work, and I see lot of people coming there to buy alcohol.”*—18-year Dalit Male

### Causes of suicide

Domestic violence, break-ups in romantic relationships, and academic exam pressure were the top three causes of suicide named. High rates of domestic violence resulting from alcohol use were reported in the LTIs, which in turn was described to be the leading cause of suicide. The participants also described their difficulty in coping with relationship problems and immense pressure they get to do well in their school leaving certificate (SLC) exams, which occur at the end of 10th grade and are the major determinant of admittance to further education.

*“My father drinks alcohol and beats my mother. I get stressed about it and cannot concentrate in school too. So, I think that it is better to die then live like this.”*—16-year-old Dalit Female

*“I think when I don’t study well, how will I become a nurse, and when I don’t become a nurse, how will I live my life? Also, I won’t be capable to do other works, so I feel like it is better to die than to live.”*—16-year-old Dalit Female

## Discussion

**Fig. 2:**
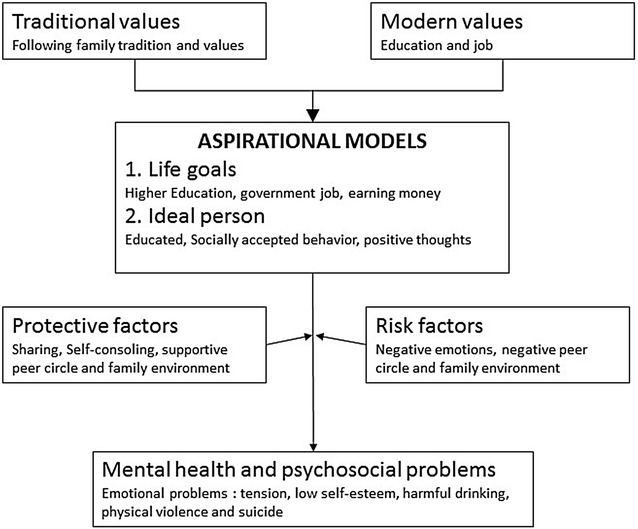
Adolescent aspirational model. Graphical model synthesizing the findings related to aspirations, emotional states, and coping strategies. The model shows how adolescents build their aspirations through the combination of traditional and modern values and how these are connected to their mental health. The figure also illustrates the different protective and risk factors

Utilizing a mixed-methods approach, we conducted qualitative life trajectory interviews and administered a card sorting task to elucidate aspirational models among adolescents in rural northwestern Nepal. We developed a framework to integrate the qualitative and quantitative findings to understand adolescent aspirational models (Fig. 2). We found that education was the most highly valued attribute of ideal persons. Educational attainment received higher prioritization by *Dalit* castes and *Janajati*, whereas *Brahman* caste youth gave education less priority. Poverty was identified as the greatest barrier to achieving life goals among all groups. The most common distressing emotion was ‘*tension’*. Girls reported ‘*tension’* more frequently than boys, and girls were most likely to drink alcohol because of ‘*tension*’. Sharing emotions and self-consoling were common behavioral response to emotional distress. *Brahman* youth were more likely to endorse coping with emotions by sharing their feelings with others, Boys reported drinking for social pleasure with peer groups. Domestic violence, ‘love tragedies’ and SLC exam pressure were the most common reasons for suicide.

An emphasis on education emerged as the central focus for aspirational models. This was closely connected with academic stress to do well on the SLC examination and then to use one’s academic success to achieve the goal of securing a stable government job. Less priority was given to the constructs of cultural practices and traditional jobs such as farming, especially among boys. Lietchy highlighted the conflict between being modern or traditional among Kathmandu youth [4]. Young people in Jumla seemed to struggle with pursuing identity goals of being both “modern” and “traditional” simultaneously. For example, they discussed fighting against the negative aspects of traditional practices, though the study data show that these barriers did not hinder achieving their life goals. They also admitted that traditional beliefs are not absolute, and it was necessary to balance tradition and modernity, especially with their older family members. Poverty was commonly seen as a bigger barrier than traditional values and practices. Similarly, tension among girls and peer pressure among boys were more dominant causes of harmful alcohol use than traditional practices were.

This also challenges some of our pre-existing expectations about rural youth aspirational models. We had thought that Jumla, often stereotyped as a traditional society, would have respondents emphasizing traditional rituals and practices. However, our current data showed their emphasis on education and employment and less focus on migration and marriage.

The focus on education as the major quality of an ideal person and the most sought-after life goal reinforces previous findings in a similar population in Nepal, among whom the promise of education led them towards youth radicalization and becoming child soldiers. Adolescents (especially girls) were found to join armed groups (Maoists in Nepal) because they did not see any hope of education in their community and were seeking alternative ways to become empowered and educated [47, 48]. This highlights the need to design interventions to increase educational opportunities for these populations, with a special focus on girls.

“Tension” could also be a key target for intervention. The English term, translated into Nepali as “tannab,” has already built up its own unique meaning in Nepali. In her study among mothers in Nepal, Clarke described tension as “having many thoughts in mind and being distracted, worried, despairing and unable to do work” [28]. This emotion stood out as the most complex and common difficulty for the adolescents in our study. It was also identified as the major reason for alcohol use especially among girls, who reported higher levels of tension than adolescent males. Interventions should focus on developing pathways for adolescents to cope with tension.

Sharing and self-consoling, considered as positive coping mechanisms, were the two most commonly used coping practices and reported to be the most effective. In post-conflict areas such as this, developing resiliency skills could be a key in developing positive mental health [49]. Studies among vulnerable groups in Nepal have shown that developing resiliency can have better outcomes and is feasible in the context of LMIC settings like Nepal [50]. Locally grounded community-based groups can be a potential intervention target for improving these practices. It could include groups like classrooms, child clubs and youth groups. Classroom-based interventions have already been started and practiced in Nepal, showing effectiveness among particular sub-groups. For example, one classroom-based intervention increased pro-social behavior among girls, which may be associated with enhanced use of sharing emotion distress and support with other girls [18].

Peer group interventions could be an excellent choice focusing on coping with emotions and behavioral changes. In rural Nepal, it has been found that children’s behavior problems are caused by negative peer influence and poor family environment [16]. Developing a positive peer circle is also equally important, as deviant peer groups were found to be one of the major reasons for adolescents to start using alcohol. In parallel, multi-level support and engagement are equally important. In another study conducted among children in rural Nepal, Adhikari and colleagues suggested using a similar kind of intervention that includes multi-level groups such as peer groups [16]. A peer group model combined with parents and a school support system can also be an important way to address suicide [51]. In his study among adolescents living in extremely impoverished communities, Farrell found that increased peer support reduced risk of suicide attempts [52]. Studies in LMICs have concluded that there is moderate to strong evidence of success of school-based interventions in promoting mental health of young people—enhancing their emotional and behavioral wellbeing, including improved self-esteem and coping skills [6, 53]. Peer group interventions can be conducted in a school-based setting in places like Jumla, where community-based children organizations (e.g. child clubs, sports clubs) are not as common as in other places. A school-based approach is well supported, with other studies among adolescents acknowledging its feasibility, effectiveness, and acceptability [15, 54–56]. In Nepal, interventions targeting other public health domains have demonstrated the success of peer support models among different castes [57].

In the same community where this study was conducted, dialectical behavior therapy (DBT) has been adapted for adult women with prior suicidal and other self-injurious behavior [58]. The adolescent aspirational models identified here could be used to adapt adult DBT for adolescent populations, which is a key period to intervene to prevent future suicidal behavior [8].

In Jumla, mental health support for the whole population, not only adolescents, is nearly absent. This is a problem globally. Although neuropsychiatric illnesses represent a large percentage of disability adjusted life years in LMICs, mental health services in national health systems in these countries are extremely weak [59]. People with mental health problems have the lowest rates of treatment for their health conditions, and integration into primary health care has been advocated as a potential solution [60]. Thus, it would be worthwhile to explore integration of adolescent mental health services into primary health care in Jumla. Such programs have already been implemented for adult mental health care in other rural areas of Nepal [61–63].

Studies have shown that in the context of LMICs, there is a need to identify and design interventions that are culturally relevant and sensitive to differences across caste and genders [64, 65]. Differences among the participant’s caste, gender, age and educational status will help to guide the design of culturally salient interventions and inform future research across these domains of mental health. In our study, Dalits were found to be most interested in attaining higher education but were least interested in government jobs. This reflects the Dalits’ perception that it would be difficult for them to access and fit in government jobs, which are mostly dominated by Brahman and Chhetri. Discrepancies were noted even within the higher caste group in terms of coping mechanisms. Chhetri chose to stay alone the most and not share their feelings and emotions with others. Interestingly, girls were found to be using more aggressive forms of coping than boys by venting their emotions on others. Girls were also more prone to tension and more likely to start drinking to cope with tension. These differences point to the need to avoid making assumptions about caste groups and gender when designing interventions. Salient caste/ethnic and gender features across different regions need to be assessed while designing these interventions.

### Limitations

Due to the card sorting activity’s requirement of literacy, the largest limitation of this study was the inability to include adolescents who were illiterate. Thus, to generalize the findings of this study, this limitation should be considered. Another limitation of this study is regarding the limited number of options during card sorting. Choices during card sorting were derived from the initial interviews, and the participants did not have the choice to add options that were unique. Variation in adolescent aspiration models and differences among caste and gender exists substantially between different regions and communities. Therefore, findings from this study should not be over generalized beyond Jumla without conducting ample ethnographic work in other communities to support these findings.

## Conclusion

Based on the findings from the card sorting activity and interviews, we ascertain that there is a need for a model of cultural intervention for adolescents in Jumla that focuses on developing peer/parent/school groups, education and job opportunities, self-esteem, and access to resources, as well as reducing tension, stress, alcohol use, and relationship problems. Research in Jumla has shown that there is an association between childhood stressors and adult depression through a gene-by-environment pathway [19]. Thus, it is important to intervene among these groups to reduce the burden of adult mental health problems. While traditional and cultural aspects cannot be separated from intervention, it is important to incorporate their changing patterns among the young and educated adolescents of Jumla. Pathways of sharing and resiliency should be further developed and strengthened. Focus of intervention should be equally on group as well as individual.

## Additional file

**Additional file 1.** Examples of coding queries exported from Nvivo 11.

## Abbreviations

DCP: disease control and prevention
LMIC: lower and middle income countries
HDI: human development index
CBS: Central Bureau of Statistics
VDC: Village Development Committee
WHO: World Health Organization
TPO: Transcultural Psychosocial Organization
KAHS: Karnali Academy of Health Sciences
NHRC: Nepal Health Research Council
LTI: life trajectory interview
SPSS: Statistical Package for the Social Sciences
SLC: school leaving certificate
