# Supplementary material for: Elucidating adolescent aspirational models for the design of public mental health interventions: a mixed-method study in rural Nepal
Source: Child Adolesc Psychiatry Ment Health. 2017 Dec 21;11:65. doi: 10.1186/s13034-017-0198-8 (PMC5740935; doi:10.1186/s13034-017-0198-8)
Supplement: Supplementary file 1 — Additional file 1. Examples of coding queries exported from Nvivo 11. [file 13034_2017_198_MOESM1_ESM.docx]

**Additional file 1**

Examples of coding queries exported from Nvivo 11. All the main 8 parent codes have been included.

“I” refers to interviewer and “R” refers to respondents.

| **Parent Code** | **Coding theme** | **Coding Query Examples** |
| --- | --- | --- |
| Qualities of an Ideal person | Coded for theme: *Education* | [Internals\\HL_02](c57e4644-5c6b-4459-b9d1-dc3b5a2d5e2c)  10 references coded, 10.36% coverage  Reference 1: 1.68% coverage  I-Now let's talk about life goals. In school when I was a kid the teachers used to ask me what is my life ambition and what I want to become in future. Maybe they ask you too. Have you thought about your life ambition and what to become in the future? R-Yes. I-What is it then? R-In the future I will study science and then I will become a teacher in my village. I want to open a school in the future and stand as a teacher and provide information to my brothers and sisters here regarding science in my village. |
|  | Coded for theme: *Religion and Tradition* | [Internals\\HL_02](c57e4644-5c6b-4459-b9d1-dc3b5a2d5e2c)  2 references coded, 2.02% coverage  Reference 1: 0.98% coverage  I-That's wonderful. Talking about the subject of religion and culture. About religion and culture, how will ideal person… R-They will always preserve their religion and culture and they talk about new things and qualities, they save their tradition, culture, and in comparison to others …. there is religious tolerance. I-They respect other's religion equally? R-They don't humiliate. |
| Life goals | Coded for theme: *Better Education* | [Internals\\HL_13](df85911c-4292-4346-a2d2-0d47602b4da9)  3 references coded, 6.12% coverage  Reference 2: 2.79% coverage  I-What's your aim? What ambition have you taken for your life? Like have you thought of anything to be next five or ten years? R-My parents are financially quite weak. My brothers and sisters have completed their education now and have started working. So if they look after me till my S.L.C examination then I plan to go to Kathmandu to study science there. And after that I will see what happens and do what I think is good for me. |
|  | Coded for theme: *Migration* | [Internals\\HL_05](8d25e426-bcc0-44ba-b8d1-ddee815db282)  4 references coded, 3.99% coverage  Reference 3: 0.56% coverage  I-Do you plan to stay in Jumla in future too or will you go abroad or go to Kathmandu? What plans do you have? R-I have plans to do my higher studies there and then return back here after completing my studies and work here. |
|  | Coded for theme: *Continue family tradition* | [Internals\\HL_P01](7988b8b6-0d78-459e-93d2-0d4763728b23)  1 reference coded, 0.83% coverage  Reference 1: 0.83% coverage  I-Let’s talk about your time again. When you were small, what did you aspire to become? R-To be engaged in agriculture. We didn’t receive any sort of education at that time so we were always involved in agriculture and we had increasing number of family members, had more children and had to raise them up and look after them. So, it was that way. |
| Barriers for life goals | Coded for theme: *Finance* | [Internals\\HL_06](59e3fb0e-f40b-4e99-8ad2-0d47593e3156)  1 reference coded, 1.71% coverage  Reference 1: 1.71% coverage  I: What problems do you think might arise to achieve the ambitions that you have set for your life? R: There might be some financial difficulties but my father has said that he will educate me even if he has to take loan. I: And then? R: If my father educates me then I will study too. |
|  | Coded for theme: *Different life goals with parents* | [Internals\\HL_15](3560121b-72f5-4fa6-96d2-0d4762108734)  1 reference coded, 2.41% coverage  Reference 1: 2.41% coverage  I-Okay you said about your life goals, to be a player and to get a permanent job and stay with your parents. Do you think it will be easy to achieve this or will it be difficult? R-I think it will be difficult. I-It will be difficult, right? So, what difficulties might there be to be a player? R-One must be hard-working to be a player and also your parents need to agree in your decision. Also, one should show the qualities of a player to teachers. It's not easy to play games one needs to be very hard-working. I-One thing you said is players should be very hard-working and another thing is parents don't agree in this so you must convince your parents. |
| Negative Emotions - Thoughts | Coded for theme: *Tension* | [Internals\\HL_07](54408698-d2c9-4a57-bdd2-0d475ab5d833)  2 references coded, 5.56% coverage  Reference 1: 2.16% coverage  R - Sometimes I get tensed I – About? R - If my hands and legs will be broken while running and I will become disabled. I get tensed about all these things; especially when I am running uphill and downhill. I already have hearing problem and to add on it if my legs and hands get broken and I become disabled, what will I do with my life? Who is going to take care of me and all?  [Internals\\HL_11](8f40db36-1ed6-4018-a7d2-0d475e840297)  1 reference coded, 2.44% coverage  Reference 1: 2.44% coverage  I: So even when others tell you these things you listen to them and just carry on your own. Do some negative thoughts come to you? R: No I don’t have those negative thoughts. I: Do you worry or become overcome by fear thinking if you would become a good artist or not? R: Yes, that happens. That’s my source of income so it worries me. I: What makes you worried? R: Thoughts like how to raise my younger brothers and sisters, how to feed them? We have salt and oil today and I fed them today but it will finish soon and from where will I bring food for them? How will I educated my younger brothers and sisters? They come home from school and they tell me that their copies and pens are finished and I feel stressed. Then I work and earn some money and give them. So, these things worry me. |
| Coping mechanisms | Coded for theme: *Sharing* | [Internals\\HL_13](df85911c-4292-4346-a2d2-0d47602b4da9)  1 reference coded, 1.63% coverage  Reference 1: 1.63% coverage  I-Next, regarding worrying, you feel you have come so far now and you worry what will happen in future and if your brothers will support you or not or if there will be any problem in your house. So, what do you do in times like these? R-At times like this I call my brothers immediately because I suppose they will say something to me and ask why I feel like that. So, I will say them openly that they have been supporting me for this long and that I am worried if they will leave me. And at that time my brother convinces me and asks me not to think like that. So, I will be searching for a cellphone always at times like that. |
| Causes of violence | Coded for theme:  *Alcohol* | [Internals\\HL_04](9212a843-5037-4231-b6d1-cd4b7565abae)  3 references coded, 2.98% coverage  Reference 1: 1.08% coverage  I-Does conflict occur between children and parents? R-It happens sometimes. I-In what things do they quarrel? R-Father drinks alcohol and say really negative things that angers you. I-What are those negative things? R-He scolds mother and say harsh things to her and we feel bad about it because we were born through her so we get angry because they shouldn't say those things to her and we threaten our father sometimes. But still they don't realize and drink alcohol and it's the same thing again so we get into conflict. |
| Reason for alcohol use | Coded for theme: *To have fun* | [Internals\\HL_12](a504ea67-8106-47de-8fd2-0d475f58ba0d)  3 references coded, 2.83% coverage  Reference 1: 0.34% coverage  I: So at other times? R: At other times during wedding ceremonies I drink with my friends just to enjoy.  Reference 2: 1.34% coverage  I: Why do you think people drink alcohol around here? R: Some people they drink because they are stressed or some people they have this addiction. Some drink just to enjoy among friends. I: You drink, too right? So, for what reason do you drink alcohol? R: When I am with friends I can’t reject, we just drink to enjoy when there’s wedding ceremonies or in other occasions or when there is newborn baby in the family. |
| Causes of suicide | Coded for theme: *Exam Pressure* | [Internals\\HL_10](54e6371e-bcea-4d7c-a7d2-0d475dacd126)  1 reference coded, 2.23% coverage  Reference 1: 2.23% coverage  R-I think like when I don’t study well how I will become a nurse and when I don’t become a nurse how will I live my life. Also, I won’t be capable to do other works so I feel like it is better to die than to live. I-You feel like it’s better to die than to live? Such things come to you mind? For what reasons do these feelings come to you? R-My father drinks alcohol and comes home and quarrel with my mother, he becomes stressed and quarrel. Also, I don’t understand what teacher teaches at school. |
